# Purification, Characterization of L-Methioninase from *Candida tropicalis*, and Its Application as an Anticancer

**DOI:** 10.1155/2015/173140

**Published:** 2015-11-24

**Authors:** Mohsen Helmy Selim, El-Zahraa Karm Eldin, Moataza Mahmoud Saad, El-Sayed Eliwa Mostafa, Yosrea Hassan Shetia, Amany Ahmed Hassabo Anise

**Affiliations:** ^1^National Research Centre, P.O. Box 12622, Dokki, Giza, Egypt; ^2^Faculty of Science, Ain Shams University, Cairo, Egypt

## Abstract

The aim of the present study is to purify L-methioninase from *Candida tropicalis* 34.19-fold with 27.98% recovery after ion exchange chromatography followed by gel filtration. The purified enzyme revealed a single band on SDS-PAGE gel with a molecular weight of 46 KDa. Its optimum temperature was 45 to 55 and thermal stability was 55°C for 15 min. The enzyme had optimum pH at 6.5 and stability at a pH range of 5.5 to 7.0 for 24 hr. The maximum activity was observed with substrate concentration of 30 *µ*M and Km was 0.5 mM. The enzyme was strongly inhibited by Cd^+2^ and Cu^+2^ while it was enhanced by Na^+^, Ni^+2^, and Mg^+2^ at 10 mM while Ca^+2^ had slight activation at 20 mM. In addition, the potential application of the L-methioninase as an anticancer agent against various types of tumor cell lines is discussed.

## 1. Introduction

L-Methioninase is one of few microbial enzymes with high therapeutic value since it was reported as a potent anticancer agent against various types of tumor cell lines: breast, lung, colon, kidney, and glioblastoma [1–3]. Many human cancer cell lines and primary tumors have an absolute requirement for L-methionine, an essential amino acid, to survive and proliferate [3, 4]. On the other hand, normal cells have the ability to grow on homocysteine, instead of methionine, due to their active methionine synthase [5]. Many tumor cells devoid of the active methionine synthase thus depend on external methionine supplementation of the diet [4]. Consequently, methionine is the main tumor specific target for therapeutic techniques. Thus, therapeutic exploitation of L-methioninase to deplete plasma methionine seems to be a promising strategy [6–9]. Furthermore, the limited distribution of L-methioninase as intracellular enzyme among all microbial pathogens, but not in humans, makes this enzyme a promising drug target for antibacterial, antifungal, and antiprotozoal therapies [10, 11].

In light of the importance of L-methioninase as a promising anticancer agent, the present study aims to purify L-methioninase from* Candida tropicalis*. Chemical and physical properties of pure enzyme were studied to improve its therapeutic applications. Moreover, the antitumor activity of purified enzyme against different cancer cell lines was evaluated.

## 2. Materials and Methods

### 2.1. Organism

The organism under study was isolated from Egyptian soil and identified as* Candida tropicalis* [12].

This strain which is able to grow on Modified Czapek Dox agar plates was cultivated in 250 mL conical flask containing 30 mL of Czapek Dox medium [13], enriched with 0.01% yeast extract. This preculture was incubated for 48 h at 30°C in a controlled environmental shaker (Model G.25, New Brunswick Scientific Co., Edison, USA). The preculture (2% vol/vol) was used to inoculate 250 mL conical flasks, each containing 30 mL of modified Czapek Dox medium (main culture) supplemented and enriched with 0.01% yeast extract. Cultures were incubated at 28 ± 2°C for 48 h with shaking (150 rpm). At the end of the incubation period, yeast cells were harvested by centrifugation (5000 rpm for 15 min). Cells cake of* Candida tropicalis* was preliminary treated with n-butanol for releasing highest yield of the enzyme by the method of [12] and assayed for their L-methioninase activities.

### 2.2. Methioninase Assay

L-Methioninase activity was assayed according to the method of [14] with some modifications using L-methionine as a substrate. Methanethiol produced from substrate reacted with 5.5-dithiobis-2-introbenzoic acid added (DTNB; Sigma-Aldrich) to form thionitrobenzoic acid which was detected spectrophotometrically at 412 nm. The assay mixture contained 20 mM L-methionine in 0.05 M potassium phosphate buffer, pH 7.0, 0.01 mM pyridoxal phosphate, 0.25 mM DTNB, and the enzyme cell-free extract in a final volume of 1 mL. After 10 min of incubation at 45°C, the increase in absorbance of the developing yellow color was measured at 412 nm. Controls without cell-free extract or with denaturated cell-free extract (cell-free extract was heated at 95°C for 30 min) were prepared separately. MTL amount was calculated according to a standard curve obtained with sodium methanethiolate. One unit (U) of L-methioninase was expressed as the amount of enzyme that releases 1 mM of methanethiol per minute under optimal assay conditions.

### 2.3. Protein Concentration

Protein concentration was determined by the method of [15] with bovine serum albumin as standard.

### 2.4. Partial Purification of L-Methioninase

All purification steps were carried out at 5–10°C unless otherwise stated. The buffers used through purification contained 20 *μ*M pyridoxal 5′-phosphate for the protection of enzyme activity [16]. The cell-free extract was heated at 60°C for different time intervals. After cooling on ice for 1 hr. the denatured proteins were removed by centrifugation at 5000 rpm at 4°C for 15 min [17].

### 2.5. Purification of L-Methioninase

*(1) Anion Exchange Chromatography on DEAE-Cellulose*. This was done according to the method presented by [18]. DEAE-cellulose column (50 × 2 cm) equilibrated with the 0.05 M potassium phosphate buffer (pH 6.5). The column was washed with the same buffer containing 0.12 M NaCl until the absorbance at 280 nm of the elute decreased to less than 0.05 absorbance units. The enzyme-fraction that showed the highest activity from the partial purification step was loaded on the column and then eluted with a linear gradient of 0.15–0.6 M in the same buffer. The flow rate was adjusted to 100 mL/hr. The enzyme activity and protein content of each elute were determined. The enzyme-active fractions were pooled and dialyzed against distilled water at 4°C. The enzyme purity was checked by polyacrylamide gel electrophoresis.

*(2) Sephadex G-200 Chromatography*. This was done according to the method presented by [19]. A Sephadex G-200 resin was soaked in 0.05 M sodium citrate buffer (pH 6.5) and allowed to swell. The swollen beads were poured down into chromatographic column (50 × 2 cm) and left to settle to a constant height of 45 cm without pressure. The enzyme-active sample from the DEAE-cellulose column was applied to a column of Sephadex G-200 equilibrated and eluted with 0.05 M sodium citrate buffer (pH 6.5) at a flow rate of 30 mL/hr. Eliot (5 mL fractions) was collected separately for the measurement of enzyme activity and protein content. Enzyme-active fractions were pooled and lyophilized. The enzyme purity was checked using SDS-polyacrylamide gel electrophoresis.

### 2.6. Determination of Molecular Weight by Gel Electrophoresis

The homogeneity of purified L-methioninase was checked using dissociating polyacrylamide gel electrophoresis (SDS-PAGE) and was carried out according to a protocol proposed by [20].

### 2.7. Amino Acid Analysis

Amino acid content was determined as described in [21].

### 2.8. Some Biochemical Properties of L-Methioninase Effect of Temperature on the Activity and Stability of L-Methioninase

The effect of reaction temperature on L-methioninase activity was determined by incubating the reaction mixture at different temperatures ranging from 30 to 60°C in 0.05 M sodium citrate buffer. The thermal stability of the purified enzyme was determined by preincubating the enzyme solution for up to 1 h at various temperatures (40, 45, 50, 55, 60, 65, and 70°C) in the absence of substrate. At different times (15–60 min), aliquots were removed and cooled and the residual activity was measured by the standard assay method as previously mentioned.

### 2.9. Effect of pH on the Activity and Stability of L-Methioninase

The optimum pH for L-methioninase activity was determined using 0.05 M sodium citrate (pH 4.0–7.0) and 0.05 M potassium phosphate (pH 6.5–8.0) buffers. After incubating each reaction at 45°C for 10 min, enzymatic activity was detected. The pH stability of the enzyme was determined by preincubating the enzyme solution at different pH values ranging from 4.0 to 8.0 with 0.02 mM PLP for 2 h at 4°C. At the end of preincubation time, the pH value of enzyme solution was readjusted to pH 6.5 and then residual enzyme activity was assayed by the standard method.

### 2.10. Effect of Inhibitors on L-Methioninase Activity

Compounds tested for their inhibitory effects included iodoacetate, glycine, phenylmethylsulfonyl fluoride (PMSF), Tris, and EDTA. The inhibitory effect of these compounds on enzyme activity was assessed by incubating enzyme solution with 1 and 10 mM concentrations of each compound for 20 min before addition of substrate. After preincubation time, enzymatic activity was determined under optimal assay conditions.

### 2.11. Measurement of Potential Cytotoxicity by SRA Assay

Potential cytotoxicity of L-methioninase was tested using the method of [22].

## 3. Results and Discussion

### 3.1. Purification of L-Methioninase from* Candida tropicalis* and Molecular Weight Determination

In the preceding part of this work, a crude enzyme preparation (CFE) was obtained from* Candida tropicalis* cells grown under optimized growth conditions as mentioned before. It was necessary to investigate and characterize such an enzyme activity. Therefore, in this section, a study on the purification of intracellular L-methioninase from* Candida tropicalis* was carried out. Furthermore, some physical and biochemical properties of pure enzyme were investigated.

**Figure 1:**
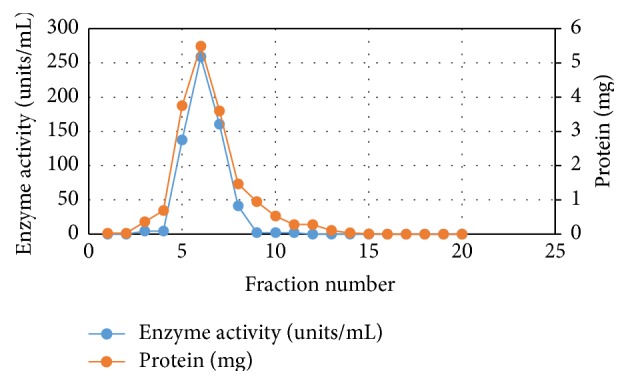
Purification of L-methioninase using DEAE-cellulose.

**Figure 2:**
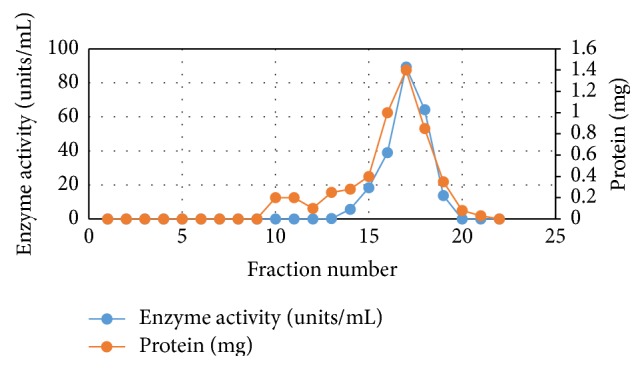
Purification of L-methioninase enzyme using Sephadex G-200.

Equal portions of CFE were purified by heating at 55°C and 60°C for different time intervals—10, 20, and 30 min. After cooling CFE in an ice bath followed by centrifugation at 5°C, three fractions were obtained for each temperature. Data presented in Table 1 indicate that following heat treatment at 60°C for 10 min gave the highest enzyme activity and enzyme recovery. Therefore, crude enzyme preparation was subjected to heat treatment at 60°C for 10 min followed by a two-step chromatographic technique-ion exchange chromatography followed by gel filtration chromatography; see Data Table 1 and Figures 1 and 2.

**Figure 3:**
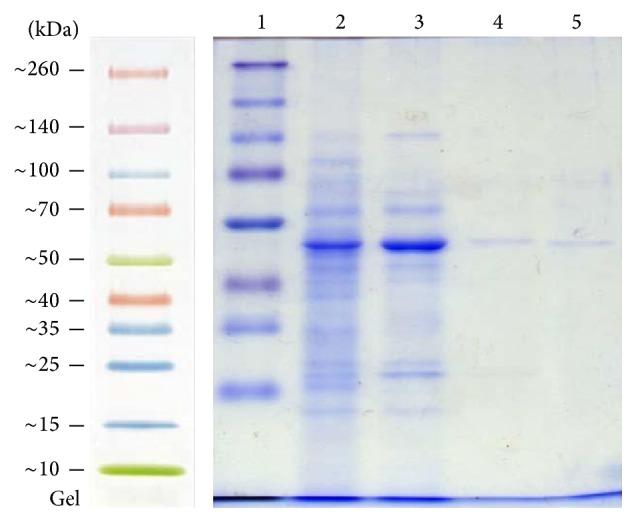
SDS-PAGE analysis of different stages during the purification of L-methioninase from* C. tropicalis*. Lane 1, molecular weight marker proteins; lane 2, crude enzyme preparation; lane 3, heat treatment fraction; lane 4, DEAE-cellulose; lane 5, Sephadex G-200. Approximate molecular mass in kilodaltons are indicated on the left.

Complete purification scheme of L-methioninase was achieved by heat treatment followed by anion exchange chromatography (DEAE-cellulose) and gel filtration chromatography (Sephadex G-100). Following heat treatment at 60°C for 10 min, the activity increased 1.5-fold compared to the crude enzyme. Furthermore, this step also decreased the overall protein content in the precipitation fraction containing the highest enzyme recovery. A successive set of purification steps was required to achieve a higher purification fold. The obtained data indicate that the purification of L-methioninase on DEAE-cellulose revealed purification fold of 24.73 with 61.4% yield. From the overall purification process, it was found that the preparation of pure enzyme can be carried out in just three purification steps, with purification fold of 43.19 and 27.98% enzyme recovery. These results are higher than those obtained by [23] for L-methioninase purified from* Brevibacterium linens* in five purification steps, including ammonium sulfate precipitation followed by several chromatographic procedures. In addition, [24] purified L-methioninase with 21% yield from* Citrobacter freundii* by heat treatment at 60°C followed by separation on DEAE-cellulose column and Sephacryl S-200HR column. Furthermore, L-methioninase was purified to electrophoretic homogeneity from* Aspergillus flavipes* 12.1-fold using ammonium sulfate precipitation followed by anion exchange and gel-filtration chromatography [25]. On the other hand, Figure 3 shows the electrophoretogram of the crude and purified L-methioninase from* Candida tropicalis* as determined using SDSPAGE. The molecular weight of the purified enzyme was estimated to be 46. In accordance with our results, the appearance of L-methionine as a single band is clear when the gel was electrophoresed under denaturing conditions ensuring the homogeneity and purity of the enzyme. The molecular mass of the purified enzyme was determined during the final stage of purification and it was estimated to be approximately 46 kDa. In accordance with our results, the purified enzyme is similar to other L-methioninases purified from different sources. As reviewed earlier, the molecular weight of L-methioninase purified from bacterial and fungal sources could range between 43 and 48 kDa [26, 27]. In addition, [23] reported that the total molecular mass of purified L-methioninase from* Brevibacterium linens* was 170 kDa, with four identical subunits, each one of 46 kDa. Also, the molecular weight of L-methioninase purified from* Citrobacter freundii* [28] was found to range from 43.0 to 45.0 kDa per subunit.

### 3.2. Amino Acid Analysis

The complete amino acid content of the purified L-methioninase enzyme was determined by analysis of protein using LC3000 amino acid analyzer (Eppendorf, Biotronik, Germany). Data not shown clearly indicate that the enzyme has high concentrations of glutamic acid followed by histidine, aspartic acid, arginine, and lysine. Furthermore, considerable concentrations of alanine, methionine, valine, serine, and isoleucine were also detected. In this respect, the amino acid composition of pyridoxal phosphate L-methioninase is clearly differentiated among various microorganisms. Reference [29] mentioned that amino acid composition of* P. putida* L-methioninase revealed that residues tyrosine, arginine, proline, leucine, glutamic acid, glycine, methionine, isoleucine, aspartic acid, histidine, lysine, and serine are commonly conserved among different L-methioninases. Reference [30] reported that several amino acid residues such as tyrosine, arginine, cysteine, lysine, and aspartic acid form active sites of* Pseudomonas putida* L-methioninase. However, L-methioninase from* Brevibacterium linens* does not have the cysteine residue and it was substituted by glycine [11, 31]. Recently it was reported that methionine, aspartic acid, histidine, and glycine were found among all the pyridoxal-phosphate dependent enzymes [32].

### 3.3. Physicochemical Properties of Purified L-Methionine

In the following part of this study, several experiments were carried out to investigate and characterize the activity of the purified enzyme in the hope of revealing some of its physiochemical properties that might be of significance in medical applications.

## 4. Time Course and Profiles of L-Methionine Reaction

Time course and profiles of L-methionine reaction (data not shown) indicate that the rate of methanethiol release increased as the reaction time increased up to 20 min; thereafter no additional hydrolysis products were liberated. Similar results were recorded for L-methioninase from* Pseudomonas putida* [17, 26, 33] where the authors studied the time course of* Pseudomonas putida* L-methionine *γ*-lyase reaction at 37°C for different times. They mentioned that the enzyme activity increased as the incubation time increased and the nonlinear increase of enzyme activity is caused by the multifunctional catalysis of L-methionine. In addition, [34] estimated L-methionine in cell-free extracts of cheese-ripening yeasts after 30 min of reaction.

### 4.1. Effect of Temperature on L-Methioninase Activity and Stability

**Figure 4:**
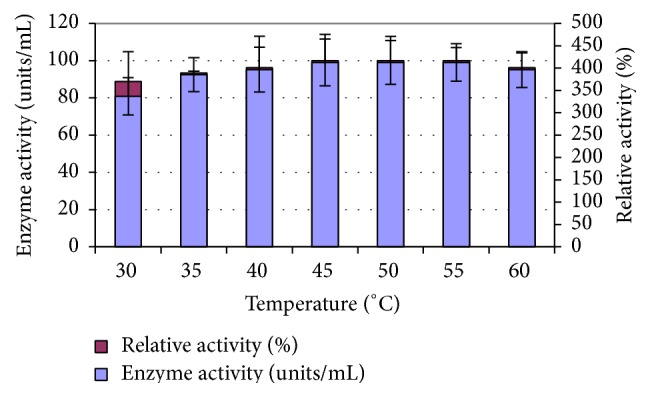
Effect of temperature on L-methioninase activity.

The optimum temperature for L-methioninase activity was determined. The temperature profile of L-methioninase was shown in Figure 4.

The optimum temperature of the enzyme activity was 45°C. Moreover, at 60°C, the enzyme retained about 96.1% of its original activity. At higher temperatures, a gradual decrease in enzyme activity was observed. This outcome may be explained by the fact that the temperature increases the reaction velocity and also affects the rate of enzyme destruction, producing a gradual fall in the concentration of active enzyme. In this finding, the optimum temperature for* C. tropicalis* L-methioninase was slightly higher than that reported for L-methioninase purified from cheese lactic acid bacteria, which was found to be 37°C [35]. Also, 37°C was the optimum temperature for L-methioninase purified from* Pseudomonas putida*. Moreover, [16] found that the activity of* Clostridium sporogenes* L-methioninase increased slightly when heated for 15 min at 50°C or for 10 min at 60°C with a rapid loss of activity after further heating at 60°C. In addition, [25] reported that L-methioninase purified from* Aspergillus flavipes* exhibited maximum activity at 35°C followed by a gradual decrease until it retained only 48.4% of its activity at 60°C. However, L-methioninase of* Brevibacterium linens* showed optimum activity at 25°C [7].

**Figure 5:**
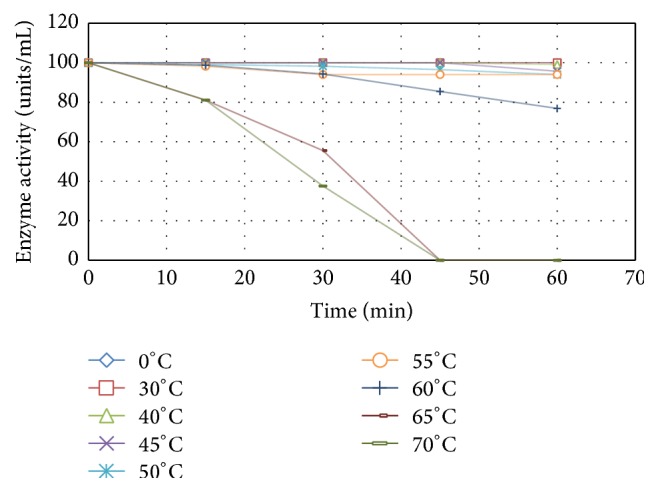
Heat inactivation kinetics of the pure L-methioninase activity.

Regarding the thermal stability of L-methioninase (Data Figure 5), there was a general agreement between optimum reaction temperatures lying in the range 45–55°C and the thermal stability of the enzyme at 55°C for 15 min. Furthermore, after heating purified enzyme at 65 and 70°C for 15 min, the enzyme still retained about 81% of its original activity. These results indicate that L-methioninase purified from* C. tropicalis* is a thermostable enzyme. In agreement with our results, [36] reported that L-methionine *γ*-lyase purified from* Aeromonas* sp. retained 82% of its original activity after incubation at 60°C for 5 min. Similarly, L-methionine *γ*-lyase of* Brevibacterium linens* NSDO739 was also stable to high temperatures as reported by [37]. On the contrary, the authors of [23] studied thermal stability of L-methionine *γ*-lyase purified from* B. linens* BL2 and they observed that the enzyme was labile at temperatures greater than 30°C.

### 4.2. Effect of Different pH Values on L-Methioninase Activity

The optimum pH for L-methioninase activity was determined by measuring activity at different pH values using 50 mM sodium citrate buffer (pH 4.0–6.5) and 50 mM potassium phosphate (pH 6.0–8.0). The enzyme activity was assayed by standard method and the results are presented in Table 2. The enzymes are generally active only over a limited range of pH values and the activity of most enzymes shows a maximum at definite pH value, that is, the optimal pH value. In this respect, the activity of L-methioninase purified from* C. tropicalis* was favored by neutral pH values with optimum pH at 6.5 and began to decrease with increasing pH value. This may be attributed to the fact that enzymes are proteins and hence any changes in the pH values can profoundly affect the ionic character of the amino acid or carboxylic groups and can therefore markedly affect the catalytic site and conformation of an enzyme. In addition, the affinity between the enzyme and its substrate may also be affected by the pH value of the reaction and the enzyme may not be saturated with substrate at pH values below or above the optimum. On the other hand, it could be noticed that the sodium citrate buffer (0.075 M) was more suitable than potassium phosphate buffer to achieve the highest enzyme activity (data not shown). This result proved that the presence of sodium ions was necessary to enhance enzyme activity. In this finding, neutral to slightly alkaline pH values were reported for L-methioninase purified from* Aspergillus flavipes* [25]. On the other hand, the optimal pH for bacterial L-methioninase has been reported to proceed at alkaline range. Reference [38] reported an optimum pH 7.2 for L-methioninase purified from* Pseudomonas ovalis*.

### 4.3. pH Stability

**Figure 6:**
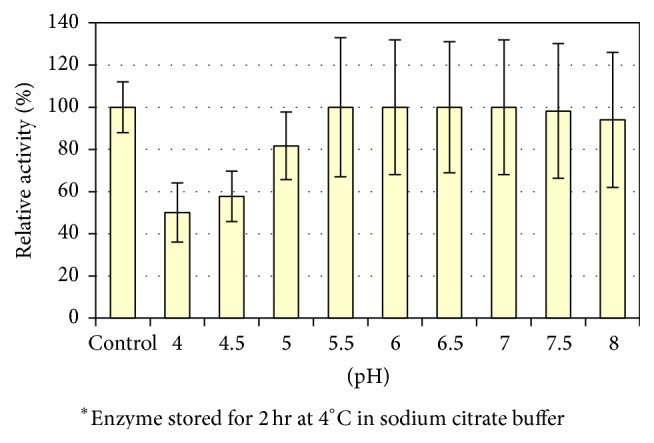
Determination of pH stability of pure L-methioninase activity.

In this experiment, enzyme solutions were stored at pH values ranging from 48 to 24 h at 4°C. Thereafter, the pH value of the enzyme solution was readjusted to pH 6.5 and added to the reaction mixture containing the substrate and the enzyme activity was then determined. The results recorded in Figure 6 indicated that the enzyme kept its activity at pH 5.5 up to pH 7.5. A slight decrease in the enzyme activity was observed at pH 8.0 while at pH 5.0 the enzyme retained about 81% of its initial activity.

Furthermore, at pH 8.0, the enzyme retained over 94% of its activity. The lower stability of the enzyme at higher or lower pH values may be caused by denaturation and hence inactivation of the enzyme protein. It is worth mentioning that the stability range of the enzyme which is higher than the pH of blood (7.4) ensures the therapeutic value of L-methioninase purified from* C. tropicalis*. The similar pH stability curve was obtained for L-methioninase purified from* Pseudomonas putida* (42.31). Additionally, the authors of [39] studied pH stability of L-methionine *γ*-lyase obtained from* Brevibacterium linens*. They found that the enzyme was stable at pH ranging from 6.0 to 8.0 for 24 h. At pH 5.5, the enzyme retained over 20% of its activity and at pH 4.0 it became inactivated.

### 4.4. Substrate Specificity of Purified L-Methioninase

**Figure 7:**
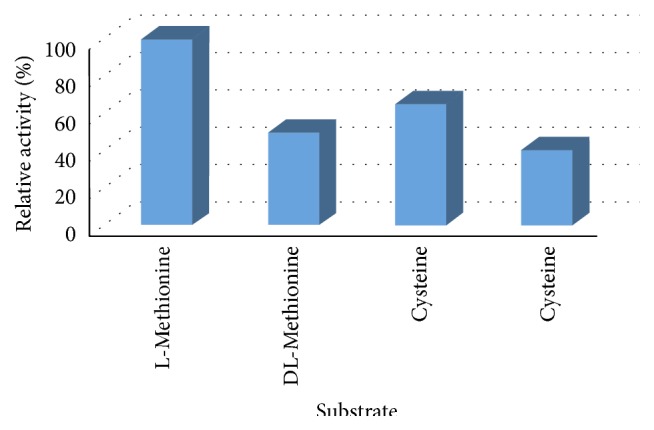
Substrate specificity of purified L-methioninase determination of kinetic parameters.

This experiment was designed to evaluate the specificity of the purified L-methioninase towards various substrates. Equal amounts (20 mM) of various substrates, namely, L-methionine, DL-methionine, cysteine, and cysteine, were added separately to the reaction mixture and incubated under optimum assay conditions. The data obtained are presented as relative activities in Figure 7. It was found that the enzyme showed the highest affinity towards L-methionine as a standard substrate. Furthermore, cysteine was degraded to 35% of the level of activity of L-methionine. On the other hand, the enzyme activities towards DL-methionine and cysteine were less than 50% and 59%, respectively, compared to that towards L-methionine. Studies on the substrate specificity of* C. tropicalis* L-methioninase revealed that the enzyme had a relative activity towards various amino acids. The enzyme exhibited high specificity for L-methionine. Additionally, L-cysteine and DL-methionine serve as effective substrates. In agreement with our results, the author of [25] examined the substrate specificity of L-methioninase purified from* Aspergillus flavipes*. The author reported that the enzyme had relative catalytic activity towards different amino acids. The enzyme exhibited high specificity for L-methionine. Furthermore, the enzyme was significantly more active on L-cysteine than on L-cystine as substrate. On the other hand,* C. tropicalis* L-methioninase is distinctly different from a bacterial enzyme in its substrate specificity where all purified bacterial L-methioninases have higher affinity towards L-cysteine compared to L-methionine [24, 40, 41]. However, [18] mentioned that L-methionine is the preferred substrate for L-methioninase purified from* P. putida*. In addition, several derivatives of L-methionine and L-cysteine serve as effective substrates.

**Figure 8:**
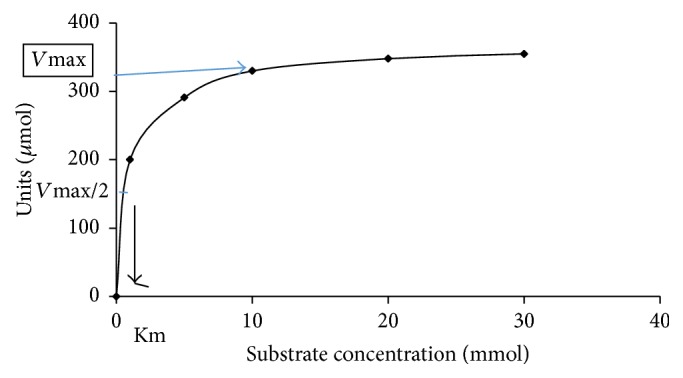
Saturation kinetics of the L-methioninase activity with the substrate.

**Figure 9:**
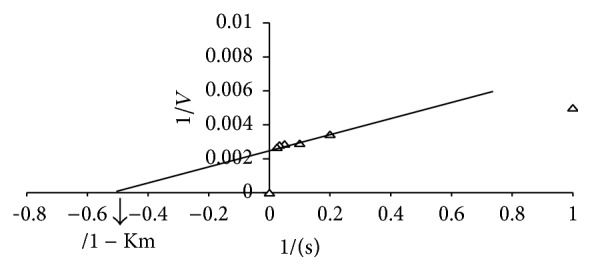
Line weaver Burk plot of the of initial velocities and substrate concentration.

In this experiment, the kinetic parameters such as Michaelis-Menten constant (Km) and maximum velocity (*V*_max_) of purified L-methioninase were determined by incubating the enzyme with different concentrations of L-methionine as a substrate in the range of 1 *μ*M to 40 *μ*M under optimum assay conditions. The apparent Km of purified enzyme was calculated from a Lineweaver-Burk plot. The Km value of L-methioninase was found to be 0.5 mM as shown in Figures 8 and 9.

Results obtained in this respect show that the enzyme showed maximal activity at a substrate level of 30 mM followed by a slight decrease at higher concentrations. This indicates that the active center of the enzyme became saturated with its substrate at concentrations above 30 mM. Moreover, the apparent Km of L-methioninase was found to be 0.5 mM indicating high affinity of* C. tropicalis* L-methioninase to its substrate and sustain its high therapeutic value. In this finding, different Km values were obtained for L-methioninase purified from various microbial sources. In this respect, L-methioninase isolated from* Pseudomonas putida* was reported to exhibit a Km of 1 Mm [42], whereas L-methioninase from* Citrobacter freundii* showed a Km of 0.7 mM [43]. In addition, the Km value for* Brevibacterium* sp. L-methioninase [23] is approximately six times higher than that observed for* Citrobacter intermedius* [44] revealing the highest specificity of the latter to L-methionine. Furthermore, it was reported that the specificity of fungal L-methioninase was higher than that observed for bacterial enzymes as clearly observed from the values of Km for L-methionine. L-Methioninase from* Clostridium sporogenes* appears to have very low specificity to L-methionine (Km value of 90 mM), suggesting its low efficiency as antitumor agent [16]. Interestingly, L-methioninase from* Aspergillus flavipes* displayed higher affinity to L-methionine, ensuring its higher therapeutic value [25].

### 4.5. Effect of Different Metal Ions and Inhibitors on L-Methioninase Activity

**Figure 10:**
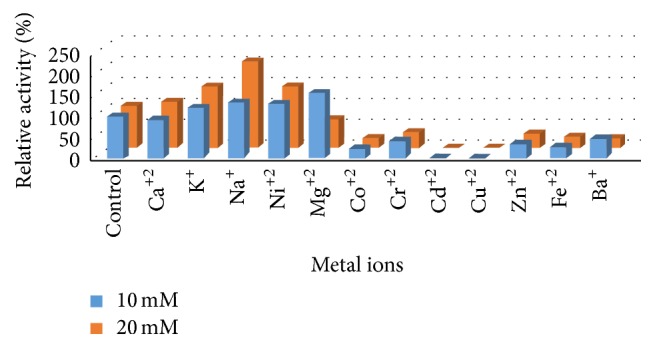
Effect of different metal ions on L-methioninase activity.

**Figure 11:**
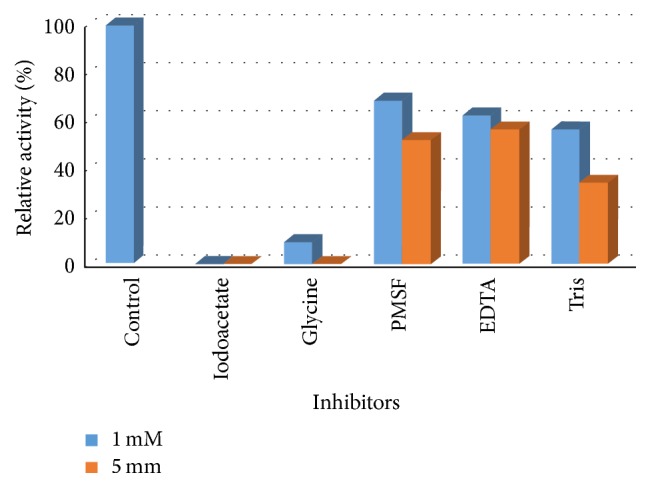
Effect of different inhibitors on L-methioninase activity.

Metal ions may serve as activators or inhibitors in numerous enzymatically catalyzed reactions. Therefore, the effect of some metal ion on L-methioninase activity was investigated (Figures 10 and 11). The results obtained in this respect indicate that Na^+^ act as a potent activator, where the enzyme activity was significantly increased to more than 200% of the original activity at a final concentration of 20 mM. Moreover, Mg^+2^, K^+^, and Ni^+2^ had an activating effect on enzyme activity. On the other hand, the enzyme activity was inhibited in the presence of Cr^+2^, Zn^+2^, Fe^+2^, Ba^+^, and Co^+2^. While Cd^+2^ and Cu^+2^ inhibited the enzyme activity completely at a final concentration of 10 Mm. It is worth mentioning that the inhibition of enzyme activity in the presence of Cu^+2^ indicated that the active site of the enzyme involved –SH group, where Cu^+2^ are known to inhibit –SH enzymes by catalyzing the oxidation of –SH to disulphide. Furthermore, significant inhibition of L-methioninase activity by EDTA, a metal ion chelator, indicates that this enzyme is a metalloenzyme. Additionally, the enzyme was completely inactivated by a thiol reducing agent such as iodoacetate. The complete inhibition of L-methioninase with this agent provides evidence for the presence of –SH group in the active sites of enzyme. In this finding, [45] mentioned that L-methioninase activity of* Brevibacterium linens* was stimulated to by Na^+^  and K^+^ and strongly inhibited by Zn^+2^, Mn^+2^, and Cu^+2^. In addition, the authors of [23] studied the effect of some inhibitors on* B. linens* L-methioninase activity. They found that iodoacetate inhibited enzyme activity at 10 mM while metal chelating agents did not influence enzyme activity. On the other hand, the activity of L-methioninase of* Lactococcus lactis* [28] and* Aspergillus flavipes* [46] was significantly reduced by sulfhydryl reagents including iodoacetic acid and 2-mercaptoethanol. Moreover, the inhibition of enzyme activity by glycine, Tris, and PMSF was also reported by [38]. It was reported that amino group of Tris molecule probably interacts with the enzyme associated PLP through a hydrogen bond and would therefore affect the enzyme activity [47].

### 4.6. Effect of Pyridoxal 5′-Phosphate Concentration on L-Methioninase

This experiment was carried out to detect the optimal concentration of PLP. The enzyme activity was assayed in standard reaction mixture supplemented with different concentrations of PLP ranging from 100 to 500 mM (data not shown). It is clear that L-methioninase activity increased gradually with the increase of PLP concentration up to 300 mM where the highest enzyme activity was obtained.

### 4.7. Cytotoxicity Test of L-Methioninase on Different Human Cancer Cell Lines

**Figure 12:**
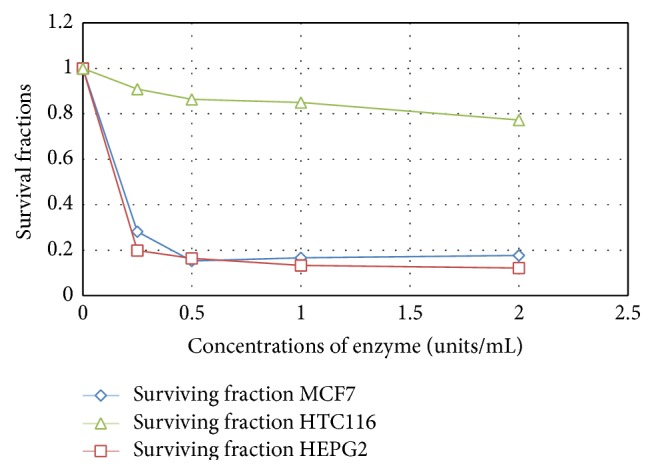
*In vitro* cytotoxic effect of partial purified L-methioninase on liver (HEPG2), breast (MCF7), and colon (HCT 116) cancer cell lines.

**Figure 13:**
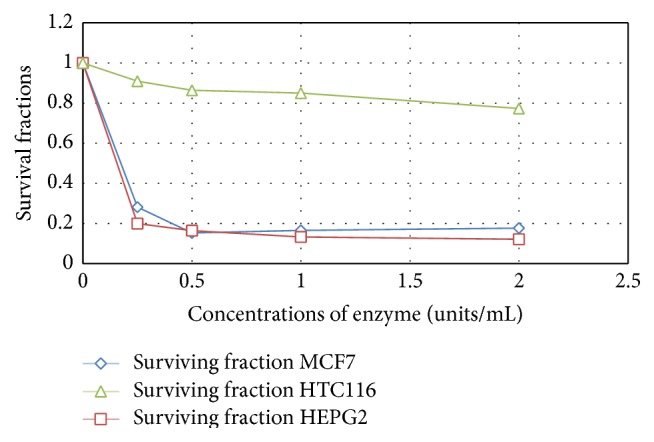
*In vitro* cytotoxic effect of pure L-methioninase on liver (HEPG2), breast (MCF7), and colon (HCT 116) cancer cell lines.

Cell-free extract containing high L-methioninase activity was obtained from* C. tropicalis* cells grown under optimum cultural and nutritional conditions. Therapeutically useful L-methioninase requires not only sufficient activity, but also an efficient method of purification [17]. Therefore, further studies were needed to purify the crude L-methioninase with the highest specific activity and good yield; see Figures 12 and 13.

*In vitro* antitumor efficiency studies of* Candida tropicalis* L-methioninase on growth of three human tumor cell lines, namely, liver (HEPG2), breast (MCF7), and colon (HCT 116), proved that the enzyme has strong antitumor activity against liver and breast cancer cell lines and this effect is concentration dependent. Moreover, sensitivity of cancer cells to enzymatic methionine depletion differed among cell lines; the breast cancer cell line was more sensitive (IC_50_ of 0.13 U/mL) than liver cancer cell line (IC_50_ 0.2 U/mL). The efficiency of L-methioninase against various cell lines was reported by many authors [2, 22, 48, 49]. In addition, the authors of [33] tested the sensitivity of cancer cell lines to L-methioninase, produced by* Pseudomonas putida*. They found that leukemia cell lines tested are more sensitive to L-methioninase than solid tumor cell lines. The IC_50_ in leukemia cell lines was less than 0.5 U/mL. On the other hand, solid tumor cell lines demonstrated variable sensitivity. The IC_50_ of lung cancer cell line was 0.8 U/mL, while the glioma cell line T98G and the breast cancer cell MCF-7 line were less sensitive to L-methioninase, with IC_50_ values of 1.5 U/mL. Furthermore, the authors of [27] evaluate the antitumor efficiency of* Aspergillus flavipes* L-methioninase against various human cell lines* in vitro*. They mentioned that the enzyme showed a powerful activity against prostate (PC3), liver (HEPG2), and breast (MCF7) cancers, with IC_50_ 0.001 U/mL, 0.26 U/mL, and 0.37 U/mL, respectively. Furthermore, our data of* Candida tropicalis* L-methioninase indicated that this enzyme was more effective against breast cancer than that produced by* Pseudomonas putida* [33] and* Aspergillus flavipes* [27].

## 5. Conclusion

This study demonstrated the ability of* Candida tropicalis* to produce L-methioninase, which has different practical applications, especially in cancer therapy. The whole production process was comprehensively optimized and the enzyme was purified to homogeneity. The purified enzyme was stable at a wide range of pH and temperature. It has high affinity to its substrate exhibiting a low Km of 0.5 mM which confirmed its high therapeutic value. Moreover, the enzyme has been found to possess significant antitumor activity against different cell lines* in vitro*. Therefore, we can say that L-methioninase treatment will provide a novel way for cancer therapy.

Until now, no publications have been reported on the purification of L-methioninase from yeast. This is the first study to include purification and characterization of L-methioninase from yeast.

## Figures and Tables

**Table 1:** Purifications steps, purification folds, and recovery yields of L-methioninase.

Purification step	Total protein (mg) ± SD	Total activity (units) ± SD	Specific activity (U/mg protein) ± SD	Enzyme recovery (%)	Purification fold
Crude enzyme	3469.2 ± 89	5243.18 ± 78	1.5 ± 0.2	100	1
Heat treatment at 60°C for 10 min	2360.9 ± 94	4876.5 ± 70	2.06 ± 0.1	93.0	1.37
DEAE cellulose fractions (3–11)	87.0 ± 21	3219.6 ± 45	37.1 ± 11	61.4	24.73
Sephadex G-100 fractions (14–20)	22.65 ± 10	1467.41 ± 38	64.78 ± 7	27.98	43.19

**Table 2:** Effect of different pH values on the purified L-methioninase activity.

pH	Type of buffer	Enzyme activity (U/ml)	Relative activity (%)
4.0	A	128.33 ± 55	38.35
	B	—	—

5.0	A	252.10 ± 60	75.35
	B	—	—

5.5	A	274.94 ± 74	82.17
	B	—	—

6.0	A	302.50 ± 69	90.41
	B	260.34 ± 80	90.92

6.5	A	334.57 ± 88	100
	B	286.35 ± 82	100

7.0	A	—	—
	B	260.79 ± 53	91.10

7.5	A	—	—
	B	245.35 ± 82	85.68

8.0	A	—	—
	B	228.0 ± 75	79.63

A: sodium citrate buffer; B: potassium phosphate buffer.
